# Contribution of the *A. baumannii* A1S_0114 Gene to the Interaction with Eukaryotic Cells and Virulence

**DOI:** 10.3389/fcimb.2017.00108

**Published:** 2017-04-03

**Authors:** Soraya Rumbo-Feal, Astrid Pérez, Theresa A. Ramelot, Laura Álvarez-Fraga, Juan A. Vallejo, Alejandro Beceiro, Emily J. Ohneck, Brock A. Arivett, María Merino, Steven E. Fiester, Michael A. Kennedy, Luis A. Actis, Germán Bou, Margarita Poza

**Affiliations:** ^1^Departamento de Microbiología, Instituto de Investigación Biomédica, Complejo Hospitalario Universitario (CHUAC), Universidad de A Coruña (UDC)A Coruña, Spain; ^2^Departamento de Microbiología y Parasitología, Universidad de Santiago de CompostelaSantiago de Compostela, Spain; ^3^Department of Microbiology, Miami UniversityOxford, OH, USA; ^4^Department of Chemistry and Biochemistry, Miami UniversityOxford, OH, USA

**Keywords:** *Acinetobacter baumannii*, biofilm, attachment, virulence, electron microscopy, secondary metabolite

## Abstract

Genetic and functional studies showed that some components of the *Acinetobacter baumannii* ATCC 17978 A1S_0112-A1S_0119 gene cluster are critical for biofilm biogenesis and surface motility. Recently, our group has shown that the A1S_0114 gene was involved in biofilm formation, a process related with pathogenesis. Confirming our previous results, microscopy images revealed that the ATCC 17978 Δ0114 derivative lacking this gene was unable to form a mature biofilm structure. Therefore, other bacterial phenotypes were analyzed to determine the role of this gene in the pathogenicity of *A. baumannii* ATCC 17978. The interaction of the ATCC 17978 parental strain and the Δ0114 mutant with A549 human alveolar epithelial cells was quantified revealing that the A1S_0114 gene was necessary for proper attachment to A549 cells. This dependency correlates with the negative effect of the A1S_0114 deletion on the expression of genes coding for surface proteins and pili-assembly systems, which are known to play a role in adhesion. Three different experimental animal models, including vertebrate and invertebrate hosts, confirmed the role of the A1S_0114 gene in virulence. All of the experimental infection assays indicated that the virulence of the ATCC 17978 was significantly reduced when this gene was inactivated. Finally, we discovered that the A1S_0114 gene was involved in the production of a small lipopeptide-like compound herein referred to as acinetin 505 (Ac-505). Ac-505 was isolated from ATCC 17978 spent media and its chemical structure was interpreted by mass spectrometry. Overall, our observations provide novel information on the role of the A1S_0114 gene in *A. baumannii*'s pathobiology and lay the foundation for future work to determine the mechanisms by which Ac-505, or possibly an Ac-505 precursor, could execute critical functions as a secondary metabolite.

## Introduction

Traditionally, *Acinetobacter baumannii* has been considered a low-virulence pathogen since its pathogenicity is influenced by the clinical condition of the patients it colonizes and infects. However, this epithet is often ignored by physicians due to its frequent and increasing occurrence as a multi-drug resistant (MDR) nosocomial pathogen around the world (Perez et al., 2008). In specific environments, such as intensive care and burn units where there is a remarkable selective antibiotic pressure, *A. baumannii* colonizes new niches because of its noteworthy ability to adapt to stressful conditions by modulating the expression of several virulence factors (Beceiro et al., 2013). Despite the importance of this microorganism, only a few virulence factors have been described to date (McConnell et al., 2013). This situation now can be addressed because of the development of inexpensive and convenient high-throughput sequencing methods, which have led to the description of numerous *A. baumannii* genomes and have facilitated the comparative analysis of entire genomes (Merino et al., 2014; Álvarez-Fraga et al., 2015; Ou et al., 2015). Furthermore, improved strategies for functional analysis of bacterial genes and the use of relevant animal models have provided novel insights into the virulence traits of this pathogen, which could lead to potential targets for the treatment of human infections (McConnell et al., 2013).

The inhibition of bacterial functions involved in quorum sensing, adhesion, colonization, iron acquisition and/or resistance to host defenses are possible strategies that could be used to fight bacterial infections, particularly those caused by MDR pathogens (Escaich, 2010). In the case of *A. baumannii*, one such strategy includes targeting capsular polysaccharides that have been identified as a virulence factors; capsule-deficient strains showed lower pathogenicity in a rat model (Russo et al., 2010). The acinetobactin-mediated iron acquisition system and related iron-mediated metabolic functions also play a role in *A. baumannii* ATCC 19606^T^ virulence as assessed using *ex vivo* and *in vivo* infection models (Gaddy et al., 2012; Zimbler et al., 2012, 2013). The ability of this pathogen to attach to different types of surfaces is also essential for its spread within the hospital environment and among patients as well as to colonize host tissues and medical devices. The outer membrane protein A (OmpA) and the biofilm-associated protein (Bap) are critical in host-pathogen interactions as well as in the interaction of bacteria with abiotic and biotic surfaces including human epithelial cells and neonatal keratinocytes (Choi et al., 2005, 2008; Iacono et al., 2008; Loehfelm et al., 2008; Kim et al., 2009). The *A. baumannii* ATCC 19606^T^ Type I pili assembled by the CsuA/BABCDE usher-chaperone assembly system were the first cellular appendages shown to be crucial for adherence and biofilm formation on abiotic surfaces under different experimental conditions (Tomaras et al., 2003). Our group previously identified the gene A1S_1507, which is part of a second Type I pili cluster, the disruption of which caused a significant decrease in biofilm formation by *A. baumannii* ATCC 17978 (Rumbo-Feal et al., 2013).

Comparative transcriptional studies of *A. baumannii* ATCC 17978 planktonic and sessile cells showed that expression of the A1S_0114 gene had the highest fold-change in biofilm-associated cells as compared to planktonic cells (Rumbo-Feal et al., 2013). Accordingly, deletion of this predicted gene led to a substantial decrease in biofilm formation (Rumbo-Feal et al., 2013). Random transposon mutagenesis of *A. nosocomialis* M2 resulted in the isolation of the M2-2 and M2-11 derivative mutants, which displayed a significant reduction in surface motility and harbored insertions in the *A. baumannii* ATCC 17978 A1S_0113 and A1S_0115 orthologs (Clemmer et al., 2011). Further RNA-Seq analysis and A1S_0112-*lacZ* fusion assays showed that the expression of the A1S_0112-A1S_0118 genes in *A. nosocomialis* M2 is transcriptionally activated by an AbaI-dependent quorum-sensing pathway (Clemmer et al., 2011). More recently, random insertion mutagenesis of the *A. baumannii* ATCC 17978hm, a hyper-motile derivative that harbors an IS insertion within the *hns*-like gene (Eijkelkamp et al., 2013; Giles et al., 2015), resulted in the isolation of the A1S_0112::Tn and A1S_0115::Tn mutants (Giles et al., 2015). Both mutants displayed no surface motility and a significant reduction in pellicle formation with an increased biofilm formation phenotype, observations suggesting a further link between motility and biofilm/pellicle formation (Giles et al., 2015). Based on all these observations, it was proposed that the A1S_0112-A1S_0118/0119 genes constitute a seven- or eight-gene operon, which is predicted to be involved in the biosynthesis of an uncharacterized secondary metabolite, such as a non-ribosomally synthesized lipopeptide (Clemmer et al., 2011; Eijkelkamp et al., 2013, 2014; Giles et al., 2015). Interestingly, this operon has been identified in many of the available genomic sequences of *A. baumannii*, including MDR strains (Adams et al., 2008; Iacono et al., 2008; Zhu et al., 2013), with the exception of the nonpathogenic *A. baumannii* SDF strain (Vallenet et al., 2008).

In this report we have established that the *A. baumannii* ATCC 17978 (referred to as 17978 in the rest of this work) A1S_0112-A1S_0119 gene cluster is indeed a polycistronic operon that includes eight genes predicted to code for proteins with functions involved in the production of bacterial secondary metabolites. Furthermore, a 17978 A1S_0114 isogenic deletion derivative (Δ0114) showed a significant reduction in cell adherence and virulence, as confirmed using three animal models. Mass spectrometry analysis of spent culture supernatants showed that the deletion of the A1S_0114 gene is associated with the absence of acinetin 505 (Ac-505), a 505-Da lipopeptide in which a hydroxylated-C_15_ acyl moiety is linked to both a Gly and a Cys-Gly containing moiety and has a non-standard peptide linkage. These observations indicate that the A1S_0114 gene could play a critical role in the pathobiology of *A. baumannii*, knowledge that could aid in the design of alternative therapeutic tools needed for the treatment of infections caused by emerging MDR isolates.

## Materials and methods

### Bacterial strains and culture conditions

*A. baumannii* ATCC 17978 and *Escherichia coli* strains listed in Table 1 were routinely grown or maintained in Luria-Bertani (LB) broth with 2% agar added for plates. All strains were grown at 37°C with shaking (180 rpm) and stored at -80°C in LB broth containing 10% glycerol. Swimming broth (SB) containing 10 g/L of tryptone and 5 g/L of NaCl was used for some phenotypic analyses and 0.3% of agarose were added for plates (Harding et al., 2013). When appropriate, cultures were supplemented with kanamycin (Km) at a final concentration of 50 μg/mL. Bacterial growth curves were determined in sextuplet using 96-well microtiter plates containing either LB or SB inoculated with 17978 or Δ0114 cells under the aforementioned culturing conditions over a 24-h time period (Figure S2). OD_600_ values of these cultures were recorded hourly.

**Table 1:** **Bacterial strains and plasmids used in this work**.

**Strain or plasmid**	**Relevant characteristic(s)**	**Sources or references**
**STRAINS**		
*A. baumannii*		
ATCC 17978	Clinical isolate	ATCC
Δ0114	ATCC 17978 A1S_0114 deletion derivative	This study
Δ0114.C	17978 Δ0114 harboring pWH1266-Km-0114; Km^r^	This study
Δ0114.E	17978 Δ0114 harboring pWH1266-Km; Km^r^, Tc^r^	This study
*E. coli*		
TG1	Used for DNA recombinant methods	Lucigen
OP50	Used for maintenance of *C. elegans*; Ura^−^, Str^r^	CGC
**PLASMIDS**		
pCR-Blunt II-TOPO	Cloning vector; Km^r^, Zeo^r^	Invitrogen
pWH1266	*A. baumannii* shuttle vector; Ap^r^, Tc^r^	Stiernagle, 2006
pWH1266-Km	*A. baumannii* shuttle vector; Km^r^, Tc^r^	This study
pWH1266-Km-0114	pWH1266-Km harboring A1S_0114; Km^r^	This study
pMo130	Suicide vector for construction of *A. baumannii* isogenic derivative; Km^r^, SacB, XylE	Hamad et al., 2009

*Ap^R^, ampicillin resistance; Km^R^, kanamycin resistance; Tet^R^, tetracycline resistance; Str^R^, streptomycin resistance; Zeo^R^, zeocin resistance*.

### Construction of isogenic deletion derivatives

Plasmid pMo130 (Table 1), a suicide vector containing the genes *xylE, sacB*, and a Km resistance marker, was used as described by Hamad et al. (2009). Briefly, 900–1,000 bp upstream and downstream regions flanking the genes selected for deletion in 17978 were PCR-amplified and cloned into the pMo130 vector using primers listed in Table S1. The resulting plasmid (pMo130-0114, shown in Table 1) was transformed into 17978 cells by electroporation (Rumbo-Feal et al., 2013). Recombinant colonies representing the first crossover event were selected by resistance to Km and visual detection of XylE activity following the catechol-based method (Hamad et al., 2009). Bright yellow Km resistant colonies were then grown overnight in LB supplemented with 15% sucrose and then plated on LB agar without antibiotics. The second crossover event leading to gene deletion was then confirmed by PCR using primers listed in Table S1. The Δ0114 isogenic deletion derivative of 17978 was constructed by deleting a region encompassing the A1S_0114 gene (Hamad et al., 2009).

### Complementation of the Δ0114 deletion derivative

A kanamycin resistance marker was PCR-amplified from the pCR-BluntII-TOPO plasmid (from Invitrogen) using the primers listed in Table S1. The resulting product was inserted in the *Pst*I site of the pWH1266 plasmid (Hunger et al., 1990), obtaining the pWH1266-Km plasmid. To complement the Δ0114 strain, the A1S_0114 wild type allele was PCR-amplified from 17978 genomic DNA using the primers listed in Table S1. The resulting product was cloned into the *Eco*RV and *Bam*HI restriction sites of the pWH1266-Km plasmid (Table 1). The parental A1S_0114 allele was cloned as a *Bam*HI-*Eco*RV amplicon into the pWH1266-Km under the control of the tetracycline resistance gene promoter using the primers listed in Table S1. The complementing plasmid pWH1266-Km-0114 (Table 1) was transformed into Δ0114 cells by electroporation (Rumbo-Feal et al., 2013). Transformants were selected on Km-containing plates and the presence of pWH1266-Km-0114 was confirmed by PCR using primers listed in Table S1. Δ0114 cells harboring empty pWH1266-Km were used as a negative control.

### RNA extraction and transcript quantification

Cultures of 17978 and its derivatives were grown for 48 h at 37°C in SB. RNA was extracted with the Maxwell 16 LEV simplyRNA Cells Kit (Promega). The total RNA samples were treated using the DNA-free DNA Removal Kit (Ambion). The integrity of the RNA samples was checked using an Agilent 2100 Bioanalyzer and qPCR. cDNA was obtained from RNA samples using the iScript cDNA Synthesis Kit (Bio-Rad) following the manufacturer's recommendations. The level of expression of particular genes was tested by real time PCR (qRT-PCR) using cDNA as a template and the KAPA SYBR FAST qPCR kit (Kapa Biosystems) following the instructions of the manufacturer with the primers listed in Table S1. Three independent biological replicates were each tested in triplicate. The expression level was standardized relative to the transcription level of the housekeeping gene *recA* which was established as 1. PCR reactions lacking cDNA were used as negative controls. The statistical significance of the differences was determined using a Student's *t*-test.

**Figure 1:**
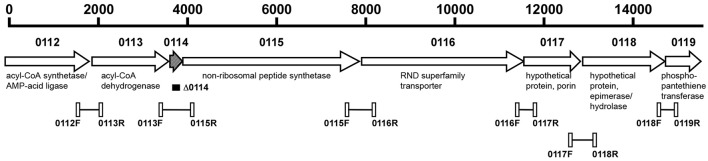
**Genetic organization of the A1S_0112-A1S_0119 operon of ***A. baumannii*** ATCC 17978**. The horizontal arrows indicate the location and direction of transcription of the predicted genes. The numbers above the arrows represent the A1S_ genes located within this operon. The black rectangle indicates the region deleted within the A1S-0114 gene represented by the hatched arrow. The connected open rectangles and the number underneath of each of them identify the primers, listed in Table S1, and the regions amplified by PCR and RT-PCR assays using genomic DNA and DNA-free total RNA, respectively. Putative functions of the corresponding proteins are shown.

To confirm the polycistronic nature of the A1S_0112-A1S_0119 gene cluster, cDNA, obtained from total RNA through reverse transcription, was used as a template in PCR reactions with Taq DNA Polymerase (New England Biolabs) using pairs of primers designed to anneal to the 3′-end of every gene and the 5′-end of the next one (Figure 1 and Table S1). Genomic DNA and total RNA without reverse transcription were used as templates for positive and negative controls, respectively, and the amplicons were detected by standard 1% agarose gel electrophoresis (Sambrook and Russell, 2001). These analyses were done in triplicate.

### LC-MS analysis of bacterial supernatants

The 17978 and Δ0114 strains were grown in static cultures and cell-free spent media was analyzed by liquid chromatography (LC)—mass spectrometry (MS) and LC-MS/MS with further internal source collision induced dissociation (ISCID) fragmentation. In brief, bacterial samples were cultured overnight in SB with shaking (200 rpm) at 37°C and used to inoculate SB at a 1:100 ratio. Then, 1-mL aliquots were dispensed into 12 × 75 mm polystyrene culture test tubes for incubation at 37°C for 48 h without shaking. Bacteria for isotopic labeling were cultured under the same conditions in unlabeled and U-^15^N, 98% and U-^15^N/^13^C, 98% Bioexpress Cell Growth Media (Cambridge Isotope Laboratories, Inc.). Samples were vortexed, transferred to microcentrifuge tubes and centrifuged at 21,000 × g to remove cells. Aliquots were prepared for LC-MS by mixing 300 μL of spent media with 1:1 volume of 100% methanol (HPLC grade) containing 0.2% formic acid. LC-MS and LC-MS/MS (ISCID) data were collected on a micrOTOF Bruker Daltonics MS with Agilent Technologies 1200 Series LC and an analytical C_18_ column (100 × 2.1 mm, 2.6 mm particle size, Phenomenex Kinetex) operated at a column temperature of 35°C with a 0.2 mL/min flow rate. Solvent A was 100% water with 0.1% formic acid and solvent B was acetonitrile with 0.1% formic acid. The elution method was a 30-min gradient from 100% solvent A to 90% solvent B in 25 min, followed by 5 min at 90% solvent B before returning to 100% solvent A for equilibration. Electrospray ionization (ESI)-MS was used in positive ion mode (*m/z* 50–2,000). Nitrogen was used as the drying gas with a temperature and nebulizing gas pressure of 190°C and 5.8 psi, respectively. The ESI capillary voltage of 4,500 V and Hexapole RF of 80 V_pp_ were used. ISCID energy of 80 eV (200 V) was applied for CID fragmentation. A sodium formate solution was employed as the external calibrant by direct infusion at the end of each run. LC-MS and LC-MS/MS (ISCID) data were analyzed using DataAnalysis 4.0 (Bruker Daltonics) software.

### High-resolution MS and MS^*n*^ analysis of Ac-505

The 17978 strain was grown for 48 h at 37°C in SB in 2 L static cultures in flat-bottom flasks after a 1:100 dilution of innoculated shaken overnight culture. After centrifugation at 5,000 × g for 20 min to remove cells, 15 g of adsorptive XAD-7 resin was added to the culture supernatant and incubated at room temperature for 3 h. The resin was removed on glass filter paper in a Büchner funnel and then washed with 1 L deionized H_2_O. The adsorbed material was then eluted with 300 ml of 100% methanol, dried on a rotary evaporator, and resuspended in 2 ml of methanol. Concentrated samples in methanol were kept at −80°C. One-ml injections were made on a BioCAD family vision Workstation equipped with a UV-detector, fraction collector, and a reversed-phase C_18_ preparative column (250 × 10.0 mm, 10 um particle size, Phenomenex Synergi Fusion-RP) operated at a 5 ml/min flow rate of the solvents A and B described above. The elution method was a gradient from 0 to 100% B in 10-column volumes, followed by an isocratic step with 100% solvent B for 5-column volumes. Eluted fractions of 1 ml were analyzed by analytical LC-MS on a Bruker micrOTOF (described above) and fractions containing purified Ac-505 were combined and dried using a stream of nitrogen.

High resolution LC-MS/MS in positive ion mode was performed at the Ohio State University (OSU) Mass Spectrometry and Proteomics Facility on a Bruker MaXis ESI Ionization Quadrupole Time-of-Flight Mass Spectrometer (ESI MaXis QTOF) with a Dionex U3000 RSLC system using a Waters Xbridge BEH C_18_ (3.5 μm, 1.0 × 100 mm) column and a gradient of H_2_O and acetonitrile with 0.1% formic acid. Additionally, direct-infusion MS/MS in negative ion mode was performed on the ESI MaXis QTOF instrument. Ultra high resolution MS/MS was performed on the OSU ESI 15 tesla fourier transform ion cyclotron resonance (15T FT-ICR) instrument with electron-capture dissociation (ECD) of the 506.3 *m/z* peak in positive ion mode by direct infusion. MS^*n*^ fragmentation in positive ion mode was measured on the OSU Bruker amaZon, which has nominal mass resolution, using direct infusion of 5-μl samples of HPLC-purified Ac-505 in 50% methanol with 0.1% formic acid.

### Bacterial adhesion to A549 human alveolar cells

A549 human alveolar epithelial cells were routinely maintained in 25-cm^2^ tissue culture flasks in Dulbecco's Modified Eagle Medium supplemented with 10% heat-inactivated fetal bovine serum and 50 U/mL of penicillin and 50 μg/mL of streptomycin (DMEM) as described before (Gaddy et al., 2009). Confluent cultures were washed, trypsinized and transferred to 24-well plates to get a monolayer of 10^5^ A549 cells *per* well. After 24 h of incubation under the same conditions, cells were washed twice with saline solution and once with modified Hank's balanced salt solution (mHBSS, same as HBSS but without glucose) following the protocol previously described (Gaddy et al., 2009). Then, the multiplicity of infection (MOI) of 10 was used; in each well 10^5^ A549 cells were infected with 10^6^ bacteria and incubated for 3 h in mHBSS at 37°C. To determine bacterial adhesion, the infected monolayers were washed three times with saline solution and then lysed in 500 μL of 0.5% sodium deoxycholate. Dilutions of the lysates were plated onto LB agar and incubated at 37°C for 24 h. Colony forming units were counted to determine the percent of bacteria that had attached to or invaded A549 cells as compared to the growth control. Six independent replicates were done. Student's *t*-test was performed to evaluate the statistical significance of the observed differences.

### Electron microscopy of biofilms formed on plastic and polarized A549 cells

Sterile plastic coverslips were placed in sterile 50-mL conical tubes and then 5 ml of LB inoculated with each strain at a 1:100 dilution were added. Inoculated tubes were incubated for 48 h at 37°C without shaking as previously described (Gaddy et al., 2009). Coverslips were removed, washed, dehydrated in ethanol, processed with a critical point drier, and sputter coated as described previously (Tomaras et al., 2003). Biofilms formed above, at and below the liquid-air interface were viewed by scanning electron microscopy (SEM) using a Zeiss Supra Gemini Series 35 V scanning electron microscope as described previously (Tomaras et al., 2003).

A549 cells were polarized on the surface of Transwell 24-well permeable inserts as recently described (Álvarez-Fraga et al., 2016). Bacteria, previously grown in LB at 37°C for 24 h in a shaking incubator at 180 rpm, were washed and resuspended in Hank's Buffered Salt Solution (HBSS; Hyclone Laboratories, Inc.). An inoculum of 10^2^ bacteria was applied to the apical surface of A549 cells by pipetting 1 μl of suspension onto the center of each membrane. The transwell plate was then incubated and maintained for 72 h at 37°C and 5% CO_2_. After 72 h, the membranes were washed with HBSS to remove secretions and unattached bacteria. The membranes were then fixed for 24 h in 4% formaldehyde-HBSS at 4°C, prepared and viewed by SEM as previously described (Tomaras et al., 2003).

### *Caenorhabditis elegans* fertility assay

Fertility assays were performed as previously described (Vallejo et al., 2015). Both the 17978 and Δ0114 strains were grown overnight in LB and then cultured at 37°C for 24 h in nematode growth medium (NG). The eggs of *C. elegans* N2 Bristol (a wild-type strain obtained from the CGC collection) were hatched in M9 medium (Vallejo et al., 2015), and worms in the first larval stage (L1) were arrested overnight at 20°C. Later, the L1 worms were added to the NG medium plates together with each bacterial strain selected for this study. One *C. elegans* worm in the last larval stage (L4) was placed on a peptone-glucose-sorbitol medium (PGS) plate individually seeded with each *A. baumannii* strain and incubated for 24 h at 25°C. The worms were transferred to new plates seeded with the same bacterial strain and the worm progeny was counted for 3 days to determine their viability. Six independent replicates were performed with each strain. Student's *t*-test was performed to evaluate the statistical significance of observed differences. Means of the differences between strains are reported.

### *Galleria mellonella* virulence assay

*A. baumannii* cells previously grown for 24 h in LB broth were collected by centrifugation and resuspended in sterile phosphate-buffered saline (PBS). Appropriate bacterial inocula were estimated spectrophotometrically at OD_600_ and confirmed by plate counting using LB agar plates. To assess virulence, *G. mellonella* survival assays were performed by injecting in triplicate 10 randomly-selected healthy final instar *G. mellonella* larvae as previously described (Gaddy et al., 2012). The dose used for each infection consisted of a 5 μl-suspension containing 10^5^ CFU (Gaddy et al., 2012). The control groups included larvae that either were not injected or were injected with the same volume of sterile PBS. The test groups included larvae infected with 17978 or Δ0114 bacteria. After injection, the larvae were incubated at 37°C in darkness, and death was assessed at 24-h intervals over 6 days. Caterpillars were considered dead, and were removed, if they displayed no response to probing. The resulting survival curves were plotted using the Kaplan-Meier method (Kaplan and Meier, 1958) and analyzed using the log-rank (Mantel-Cox) test. *P* ≤ 0.05 were considered statistically significant (SAS Institute Inc.).

### Murine pneumonia virulence assay

A pneumonia model was used to evaluate the virulence of the 17978 and Δ0114 strains. BALB/c 9- to 11-week old female mice weighing 25–30 g were intratracheally infected with ~5.5 × 10^7^ CFUs/mouse of exponentially grown cells of the 17978 parental or the Δ0114 mutant strains into groups of 10 mice. Briefly, mice anesthetized with an oral suspension of sevoflurane (Abbott) were suspended by their incisors on a board in a semi-vertical position. Correct intratracheal inoculation was confirmed by using an endoscope on the oral cavity. The trachea was accessed using a blunt-tipped needle for the inoculation of a 40-μl bacterial suspension made in sterile saline solution and 10% porcine mucin (wt/vol; Sigma) mixed at a 1:1 ratio. Dead mice in the first 4 h after inoculation were not included in the final analyses. Mice were euthanized with an overdose of thiopental sodium (Sandoz) 44 h after inoculation. Lungs were aseptically extracted, weighed, and homogenized in 1.5 ml of ice-cold saline solution in a Mixer Mill dismembrator (Retsch). Lung lysates were 10-fold serially diluted and samples were plated onto LB agar to measure organ bacterial loads. The results are shown as means of the log_10_ CFU *per* gram of lung with their standard deviations. Student's *t*-test was performed to evaluate the statistical significance of the observed differences. All mice were maintained in the specific pathogen-free facility at the Technology Training Center of the Hospital of A Coruña (CHUAC, Spain). All experiments were done with the approval of and in accordance with regulatory guidelines and standards set by the Animal Ethics Committee (CHUAC, Spain).

## Results

### Characterization of the *A. baumannii* ATCC 17978 A1S_0112-0119 operon

Previous transcriptional analysis of 17978 planktonic and sessile bacteria showed the differential expression of the A1S_0112-A1S_0118 genes, with A1S_0114 being expressed most highly by cells attached to an abiotic surface (Rumbo-Feal et al., 2013). *In silico* analysis of the chromosomal region harboring these genes indicates that it potentially codes for a polycistronic operon that includes the A1S_0119 coding region (Figure 1). This prediction is based on the observation that all putative genes are transcribed in the same direction and either overlap or are separated by intergenic regions ranging from 24 to 53 nucleotides according to reported genomic data (Smith et al., 2007) and our *in silico* analysis. The polycistronic nature of this operon was confirmed by RT-PCR analysis of total RNA using primers connecting the eight predicted genes. Figure S1 shows the detection of the predicted amplicons when total RNA was reversed transcribed and PCR amplified using the primers shown in Figure 1 and listed in Table S1, with their sizes matching those detected when total DNA was used as a template. In contrast, no amplicons were detected in any of the RNA samples that were PCR amplified without previous reverse transcription.

The analysis of 17978 genomic data showed that the 15,551-nt region harboring the A1S_0112-A1S_0119 predicted coding region is separated from an upstream *luxR* ortholog (A1S_0111) and a downstream tRNA-Gly (A1S_0120) gene by a 616-nt and a 95-nt non-coding region, respectively, with the latter gene being transcribed in the opposite direction.

### Inactivation of A1S_0114 affects bacterial interaction with abiotic and biotic surfaces

**Figure 2:**
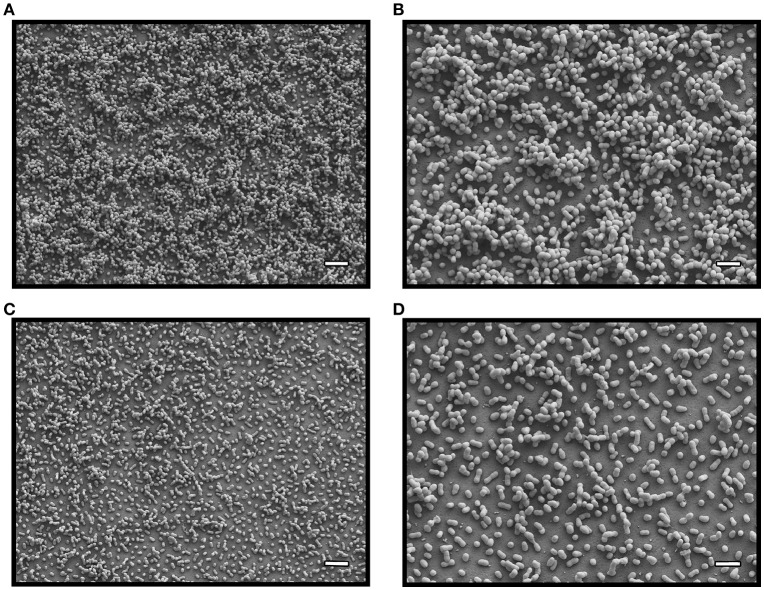
**SEM analysis of bacterial biofilms**. Biofilms formed by 17978 (micrographs **A** and **B**) and Δ0114 (micrographs **C** and **D**) cells at the liquid-air interface. Micrographs A and C were recorded at a 5,000x magnification, while micrographs **B** and **D** were collected at a 10,000x magnification. White bars: 4 μm for **A**,**C**; 2 μm for **B,D**.

In previous work we demonstrated the involvement of the A1S_0114 gene in the ability of 17978 cells to form biofilms on abiotic surfaces using crystal violet assays (Rumbo-Feal et al., 2013). This result is further supported in the present work by the analysis of biofilms formed on glass using SEM, which showed that 17978 cells attach to the abiotic surface and form multicellular structures associated with mature biofilms at the air-liquid interface (Figures 2A,B). In contrast, single or small cell clumps of the mutant derivative lacking the A1S_0114 gene (Δ0114) attached to the substratum without forming dense and three-dimensional structures (Figures 2C,D). It is important to note that the site-directed deletion of A1S_0114 did not affect the growth of the isogenic derivate when cultured either in Luria-Bertani (LB) or swimming broth (SB) without selective pressure (Figure S2).

**Figure 3:**
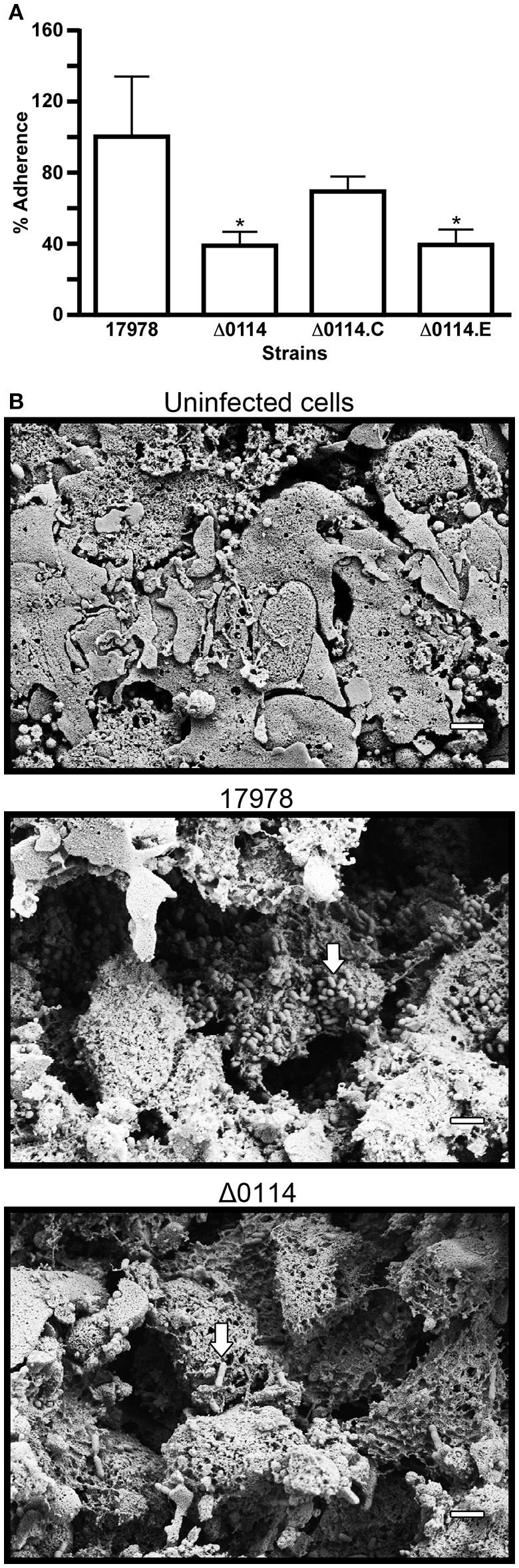
**Analysis of bacteria-human cell interactions. (A)** Quantification of the adherence of 17978 and Δ0114 bacteria either not transformed or transformed with pWH1266-Km-0114, which harbors the A1S_0114 parental allele (Δ0114.C), or the empty shuttle vector pWH1266.Km (Δ0114.E) to polarized A549 cells. Bacterial adherence is reported as the % of recovered bacteria compared to data collected with 17978, which was considered 100%. Data represent three independent replicates. Student's *t*-test was used to validate the experimental data, values are means and bars indicate standard deviation (^*^P < 0.05). **(B)** SEM analysis of A549 polarized cells either not infected (Uninfected cells) or infected with 17978 (17978) or with Δ0114 bacteria (Δ0114). All micrographs were taken at 10,000x magnification. White bars indicate the scale marks (2 μm). White arrows indicate bacteria attached to A549 cells or cell debris.

The biological effect of the A1S_0114 deletion was also tested using A549 human alveolar epithelial cells as a model since they represent a host cell that could be targeted by *A. baumannii* during the pathogenesis of respiratory infections. To test the effect of the A1S_0114 deletion on bacterial adherence, submerged A549 confluent monolayers were co-incubated with 17978 or Δ0114 bacteria for 3 h and CFU counts were determined by plating serial dilutions of tissue culture cell lysates. This approach showed that the amount of Δ0114 bacteria recovered from A549 infected cells was 60% lower than that recovered from monolayers infected with 17978 bacteria (*P* = 0.0198; Figure 3A). Electroporation of Δ0114 cells with pWH1266-Km-0114, which harbors the A1S_0114 wild type allele expressed from the tetracycline resistance gene promoter, partially restored the adherence phenotype in the Δ0114.C derivative, a phenomenon that was not observed with Δ0114.E, a Δ0114 derivative transformed with the pWH1266-Km empty vector (*P* = 0.0067; Figure 3A).

The results obtained with submerged A549 monolayers were further confirmed by infecting A549 polarized cells with either the 17978 parental or the Δ0114 mutant strains. SEM analysis of A549 samples infected with 17978 bacteria revealed extensive damage to the surfactant layer that covers the polarized cells as well as to the epithelial cells themselves when compared with non-infected polarized cell samples (Figure 3B). Furthermore, micrograph 17978 shows the presence of numerous bacteria attached to the surface of cells or cell debris clearly seen after the surfactant layer was destroyed by bacterial action. Although the infection of A549 polarized samples by Δ0114 bacteria also resulted in destruction of the surfactant layer and cell damage, it appears that the deletion of A1S_0114 results in a readily detectable reduction of bacteria attached to the polarized samples (Figure 3B).

### Role of A1S_0114 in the expression of genes involved in adherence and biofilm biogenesis

The observation that the deletion of A1S_0114 significantly affects bacterial adherence and biofilm biogenesis, prompted us to examine the differential expression of genes known or predicted to be involved in these *A. baumannii* cellular functions, including *ompA* and the *csuA/B* gene of the *csuA/BABCDE* pili assembly system (Tomaras et al., 2003; Gaddy et al., 2009; Cabral et al., 2011), and the genes A1S_0690, A1S_1510, and A1S_2091 that could be involved in bacteria-surface interactions (Rumbo-Feal et al., 2013; Eijkelkamp et al., 2014; Nait Chabane et al., 2014; Álvarez-Fraga et al., 2016). The comparative qRT-PCR analysis of total RNA isolated from 17978 and Δ0114 bacterial cells showed that the transcription of *csuA/B* is 5-fold increased in the Δ0114 mutant when compared with the parental wild-type strain (Table 2). In contrast, the transcriptional expression of the A1S_0690, A1S_1510, and A1S_2091 genes were significantly reduced, with A1S_2091 and A1S_1510 showing the highest (12-fold) and lowest (1.5-fold) changes, respectively. Deletion of A1S_0114 also caused a small (1.6-fold) but significant reduction in the transcription of *ompA*.

**Table 2:** **Expression level of genes ***csuA/B***, A1S_2091, A1S_1510, A1S_0690, and ***ompA*** in ***A. baumannii*** ATCC 17978 and its mutant derivative 17978 Δ0114**.

**Gene^*a*^**	**ATCC 17978**	**17978 Δ0114**	***P*-value**
*csuA/B*	0.36 ± 0.14	1.81 ± 0.40	0.004
A1S_2091	2.64 ± 0.41	0.22 ± 0.04	0.0005
A1S_1510	0.86 ± 0.10	0.56 ± 0.04	0.0085
A1S_0690	8.48 ± 0.92	3.07 ± 0.38	0.0007
*ompA*	1.23 ± 0.14	0.76 ± 0.08	0.0071

a *The expression level of each gene was determined with respect to the expression level of recA, which was defined as 1*.

*Values are means ± standard deviation. P-values indicate significant differences as determined by a Student's t-test*.

### A1S_0114 plays a role in virulence

**Figure 4:**
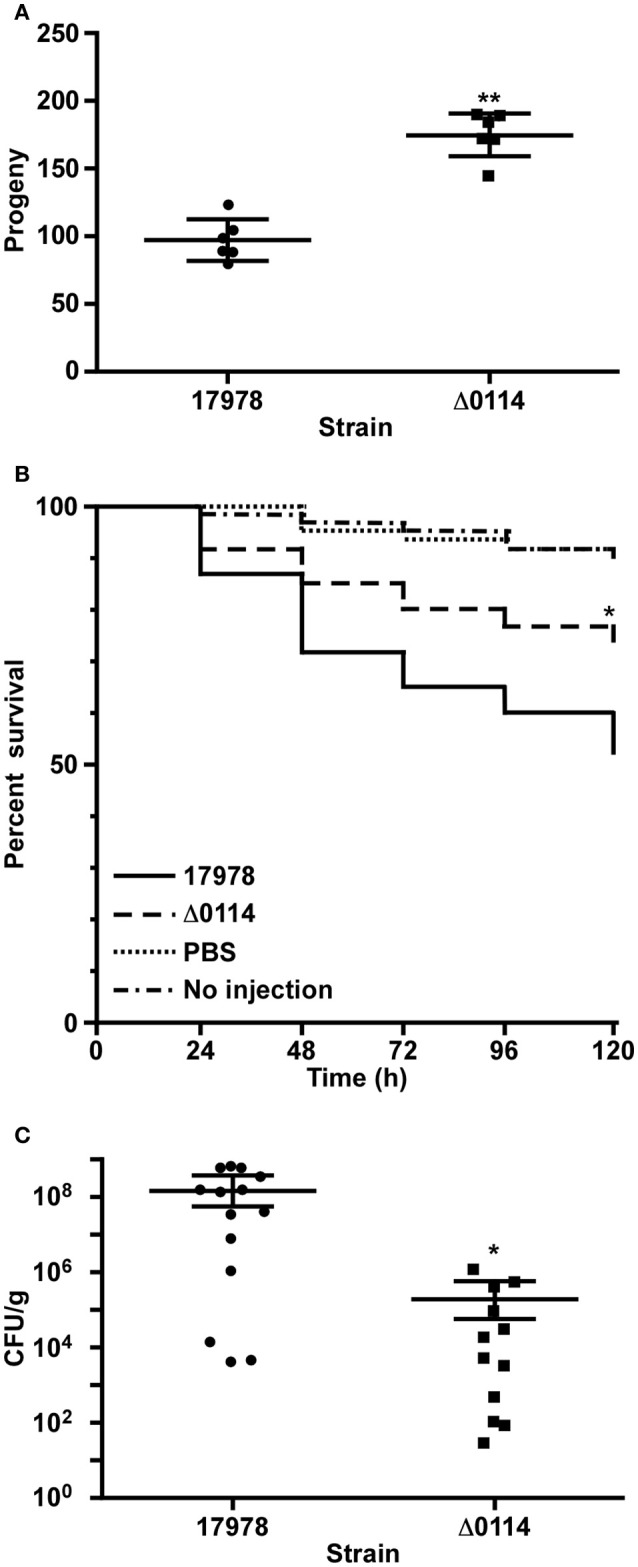
**Involvement of A1S_0114 in ***A. baumannii*** virulence**. The virulence of the 17978 and Δ0114 strains was tested using *C. elegans* fertility and *G. mellonella* killing assays and a mouse pneumonia model. **(A)** *C. elegans* strain N2 was fed with 17978 or Δ0114 bacteria for 3 days counting progenies daily using six independent biological replicates. Each dot represents the worm progeny counted during 3 days. **(B)** *G. mellonella* caterpillars (*n* = 60 *per* group) were injected with 17978 or Δ0114 bacteria and death was determined daily for 5 days while being incubated 37°C in darkness. Caterpillars not injected or injected with the same volume of sterile PBS were used as negative controls. Log-rank (Mantel-Cox) tests were done to statistically validate experimental data. The asterisk in panel **(B)** indicates a *P* < 0.05 when the mutant and the wild type strains were compared. **(C)** Mice (*n* = 10 *per* group) were infected with ~5.5 × 10^7^ exponentially growing cells of the 17978 parental strain or the Δ0114 mutant *via* intratracheal intubation. The number of bacterial cells in lung homogenates was determined 24 h post infection. Student's *t*-test was used to validate experimental data shown in panels **(A,C)**. Values in panels **(A,C)** represent means and bars indicate the standard deviation (^*^P < 0.05; ^**^P < 0.001).

The role of the A1S_0114 gene in the virulence of 17978 was assessed with a fertility assay using the *C. elegans* model, a survival assay using the caterpillar *G. mellonella* and a mouse pneumonia model, all of which have been previously used to examine the virulence of *A. baumannii*, particularly that of the 17978 strain (McConnell et al., 2013). *C. elegans* fertility assays showed that the total number of viable eggs was almost twice as high when worms were infected with Δ0114 when compared with 17978 (Figure 4A), with the difference between these two isogenic strains being statistically significant (*P* < 0.0001). Similarly, infection of *G. mellonella* larvae showed that *ca*. 50% of them died 5 days after being injected with 17978 (Figure 4B). This value was significantly different (*P* = 0.024) from that obtained with non-injected animals or animals injected with sterile PBS, which were used as negative controls. Although the infection of caterpillars with Δ0114 showed a killing rate that was significantly higher than the negative controls, the virulence of the mutant was significantly attenuated when compared with the parental strain (*P* = 0.025). Finally, the ability of the Δ0114 mutant to establish infection in an experimental murine model was evaluated. This model showed that lungs from mice infected with Δ0114 displayed a significantly lower bacterial burden than those from animals infected with 17978 (*P* = 0.0165) after 44 h of intratracheal infection (Figure 4C).

### Identification and characterization of the secondary metabolite Ac-505

**Figure 5:**
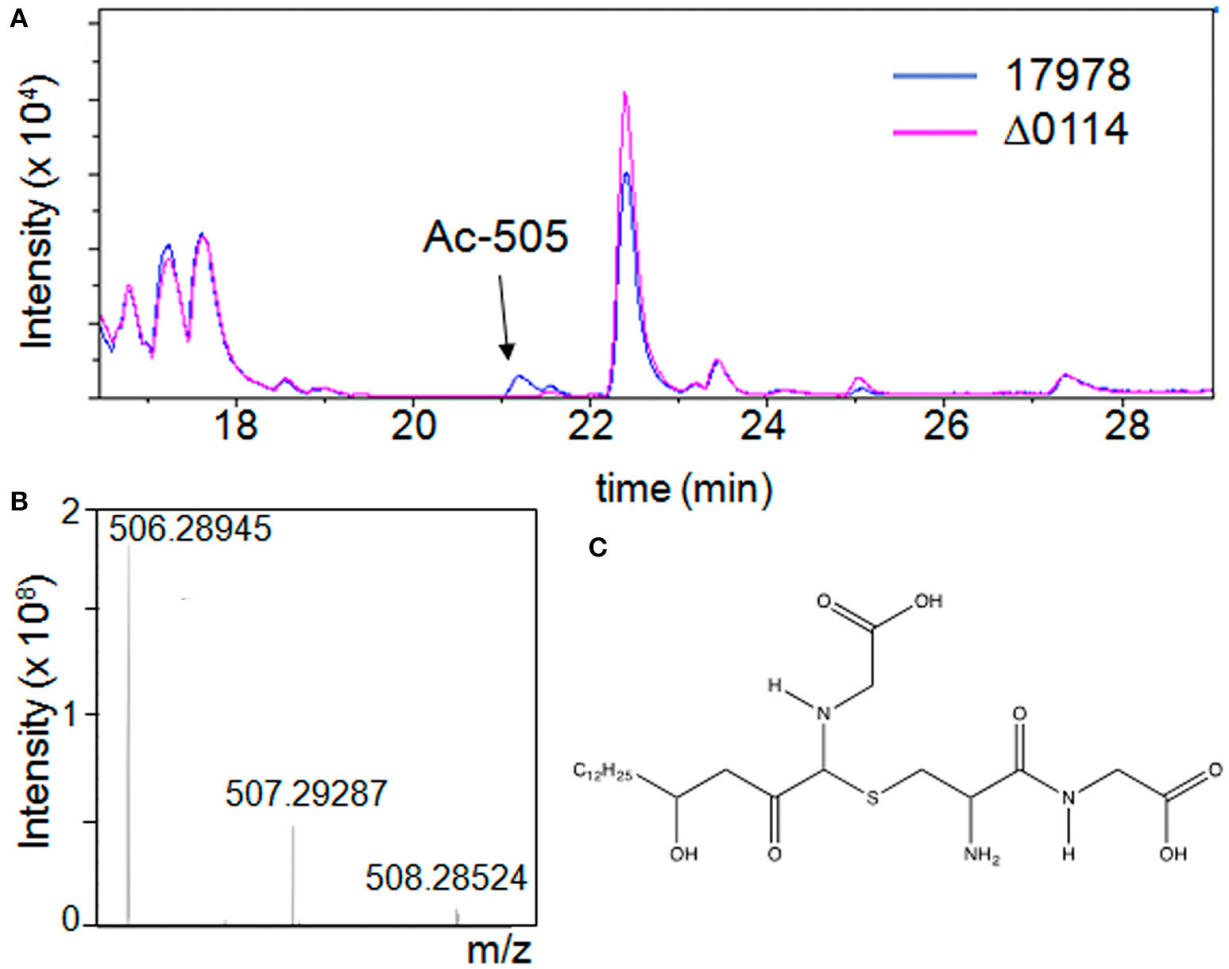
**Detection of Ac-505 in bacterial culture supernatants**. **(A)** LC-MS chromatogram of culture supernatants from cells of the parental strain (17978) in blue and the A1S_0114 deletion derivative (Δ0114) in pink grown in static SB. The arrow indicates a compound (peak Ac-505) not present in the mutant profile. **(B)** Ultra high-resolution mass spectrum of HPLC purified Ac-505 by ESI FT-ICR showing the isotopic distribution of the [M+H]^+^ ion. **(C)** Proposed structure of Ac-505.

The observation that the A1S_0114 gene plays a critical role in the pathobiology of *A. baumannii* prompted us to initiate a search for compounds that were produced by cells of the 17978 parental but not by cells of the Δ0114 mutant derivative. Liquid chromatography/mass spectrometry (LC-MS) analysis in positive ion mode of swimming broth (SB) static culture supernatants harvested at 48 h post inoculation showed the presence of a single peak with a retention time of ~21 min and a molecular weight of 505.28 Da (*m/z* 506.29 [*M*+H]^+^), which was consistently present in 17978 samples but not detected in samples from the Δ0114 deletion derivative (Figure 5A). Based on this finding, we have named this compound acinetin 505 (Ac-505). Time course studies of SB cultures harvested every 24 h for 5 days (data not shown) showed a maximum amount of Ac-505 at 48 h post inoculation that leveled off. Further analysis of Ac-505 isolated and purified from 2 L of static SB culture supernatant showed that its molecular formula is C_23_H_43_N_3_O_7_S based on ultra high-resolution 15T FT-ICR MS (*m/z* 506.28945 [*M*+H]^+^, calculated 506.28945) as shown in Figure 5B. The number of predicted nitrogen and carbon atoms was further confirmed by growing 17978 cultures in unlabeled, and ^15^N- and ^15^N/^13^C-labeled media. LC-MS analysis of these culture supernatants confirmed the incorporation of 3 nitrogen and 23 carbon atoms in Ac-505 (*m/z* 506.3, 509.3, and 532.3 [*M*+H]^+^, respectively). Additional high-resolution mass spectrometry (HRMS) analyses of FT-ICR ECD and MaXis QTOF MS/MS data are reported in Tables S2, S3 and Figures S3, S4C,D, S5, S6. Nominal mass LC-MS^*n*^ fragmentation data from the Bruker amaZon were also used in the structural interpretation and two of the 40 spectra are shown in Figures S4A,B. Positive ion mode data were used to confirm neutral losses of water, two C-terminal glycines, cysteine, and cysteine-glycine as seen in Figures S4A,C. The MS/MS fragmentation data in both positive and negative ion mode correspond to data for other Cys-Gly conjugated compounds (Levsen et al., 2005). Specifically, the negative ion with 143.05 *m/z* is characteristic for detection of Cys-Gly conjugates (Dieckhaus et al., 2005). By analysis of the MS^*n*^ and FT-ICR fragmentation data, there is a neutral loss of water followed by loss of Cys-Gly and a second water leaving the remaining ion with the formula of C_18_H_32_NO3^+^ (310.2 *m/z*) as seen in Table S2 and Figure S4C. Further neutral loss of C_2_H_5_NO_2_ from the 310.2 *m/z* ion matches a terminal glycine loss and the remaining 235.2 *m/z* ion has the formula C_16_H_27_O^+^ (Table S2). The fragmentation patterns in the positive ion mode MS^*n*^ data for 310.2 and 235.2 *m/z* (shown in Figure S4B) had characteristic neutral losses of at least six 14 *m/z* corresponding to CH_2_ losses, indicating a hydrocarbon chain of at least C_6_. A neutral loss of C_14_H_28_O from the 504.3 *m/z* parent ion was observed by MS/MS in negative ion mode as is shown in Figure S3C. Taken together, the MS data indicate that the structure of Ac-505 contains a Cys-Gly that is connected to the rest of the molecule via a sulfur linkage through the Cys side chain in a non-standard peptide linkage as shown in Figure 5C. The attachment of the Cys-Gly to the α-carbon of a Gly-containing moiety results in a so-called α-thio linkage. The Gly-containing moiety is connected to a hydroxylated acyl moiety of 15 carbons with a linkage that is neither *N*- or *O*-linked. The MS data supporting the structure shown in Figure 5C are detailed in Tables S2, S3 and Figures S4–S6.

## Discussion

*A. baumannii* has emerged as a pathogen with a remarkable ability to adapt and persist in response to a wide range of extracellular stimuli (Fiester and Actis, 2013). Such capacity relates to its genetic plasticity and the acquisition of genes coding for virulence-associated functions by horizontal gene transfer mechanisms. While some of these genes code for functions clearly related to bacterial pathogenicity (McConnell et al., 2013), there are other genes that code for potential virulence-associated traits whose mechanisms of action and biological roles are not fully understood. Among these genes, there is a gene cluster that was identified by our previous work as well as by other investigators using different *Acinetobacter* clinical isolates.

Study of the *A. baumannii* ATCC 17978, *A. baumannii* ATCC 17978hm and *A. nosocomialis* M2 strains resulted in the identification of the A1S_0112-A1S_0118 gene cluster while the analysis of the *A. baumannii* AB307-0294 isolate identified the ABBFA_003406-ABBFA_003399 cluster (Clemmer et al., 2011; Rumbo-Feal et al., 2013; Allen and Gulick, 2014; Giles et al., 2015). Importantly, these gene clusters code for cognate proteins that are highly related. Based on the nucleotide structure and the coding nature of the genes contained within this cluster of orthologous genes, it was hypothesized that it is either a 7- or 8-gene operon (Clemmer et al., 2011; Rumbo-Feal et al., 2013; Allen and Gulick, 2014; Giles et al., 2015). Transcriptional data presented in this report proved that this cluster is indeed a single 8-gene polycistronic transcriptional unit that encompasses the A1S_0112-A1S_0119 coding regions. This genetic structure explains the differential transcription reported for the A1S_0112-A1S_0118 genes in planktonic cells as compared to sessile cells (Rumbo-Feal et al., 2013). Our previous transcriptomic and mutagenesis studies also showed that site-directed deletion of A1S_0114, one of the highest transcribed genes in sessile cells when compared to exponential- and stationary-phase planktonic bacteria, caused a drastic reduction in biofilm biogenesis (Rumbo-Feal et al., 2013), a result that has been further confirmed in this work. The Δ0114 mutant not only forms less biofilms on plastic (Figure 2), but also shows less adherence when incubated with submerged or polarized human alveolar epithelial cells (Figures 3A,B). Interestingly, although the infection of polarized A549 cells with the Δ0114 mutant resulted in less biofilm formation on the surface of polarized cells, it did not result in an appreciable difference in the damage of the eukaryotic cells and the mucin layer that covers them when compared with samples incubated with the 17978 parent strain under the same experimental conditions (Figure 3). This observation suggests that A1S_0114 differentially affects distinct host-pathogen interactions that ultimately lead to the pathogenesis of respiratory infections caused by this pathogen.

If the phenotypes described above were to be pili mediated, as is the case of many bacteria, we would expect a correlation between the presence of an active A1S_0114 gene and the expression of genetic traits potentially involved in pili production and assembly. Accordingly, deletion of A1S_0114 significantly reduced the transcription of the A1S_0690, A1S_1510, and A1S_2091 genes (Table 2). A1S_0690 is a component of the A1S_0690-A1S_0695 operon, which codes for a predicted FilF-like Type III pili highly produced by pellicle cells (Marti et al., 2011; Nait Chabane et al., 2014); A1S_1510 is a component of the A1S_1507-A1S_1510 operon that codes for a predicted Type I pili, the production of which is controlled by iron and H-NS (Eijkelkamp et al., 2011a); and A1S_2091 is a component of the A1S_2088-A1S_2091 operon, which codes for an uncharacterized Type I pili also detected in pellicle cells (Nait Chabane et al., 2014; Álvarez-Fraga et al., 2016). Also, changes in biofilm biogenesis and adherence may reflect the fact that these responses could be mediated by the products of the *ompA* and *csuA/B* genes as described before (Tomaras et al., 2003; Gaddy et al., 2009). The expression of OmpA was reduced in the decreased biofilm former Δ0114 mutant, an observation that is in agreement with previous work (Gaddy et al., 2009). However, the biological relevance of the differential transcription of the *csuA/B* gene belonging to the *csuA/BABCDE* operon is not clear at the moment. It has been reported that this chaperone-usher pili assembly system may not be active in the 17978 strain due to a single base-pair insertion in *csuB* (Eijkelkamp et al., 2011b), although the CsuC and CsuD proteins have been detected as overproduced proteins in 17978 pellicle cells (Marti et al., 2011).

This report also provides the first experimental evidence that the A1S_0114 gene contributes to the virulence of *A. baumannii* when tested with three different experimental infection models already used to study *A. baumannii*'s virulence. It is apparent from our data that the marker-less deletion of A1S_0114, caused a significant reduction in virulence independently of the experimental infection model used to test this phenotype (Figure 4), without affecting the overall growth of this derivative in rich medium when compared to the 17978 parental strain (Figure S2).

It is interesting to note that a genome wide analysis using a random transposon mutant library containing 150,000 unique insertion derivatives, which included insertions in all components of the A1S_0112-A1S_0119 operon, showed that, in contrast to our observations, mutations within this operon did not affect the persistence of 17978 in the lungs of infected animals (Wang et al., 2014). Collection of samples at different times after infection (24 vs. 44 h) and the use of different mice strains (BALB/c vs. C57BL/6) could explain the disagreement between our data, collected using mutants generated by a site-directed approach, and that recorded with random transposon insertion derivatives. Similarly, different infection routes (pneumonia vs. bloodstream infection) and mice strains (BALB/c vs. leukopenic CBA/J) used to identified 17978 genes required for bacterial survival in the bloodstream of infected mice could explain the observation that mutants with random insertions in all A1S_0112-A1S_0119 genes did not meet the threshold to be considered critical for bacterial survival and no insertion within A1S_0114 was reported (Subashchandrabose et al., 2016). Genetic differences between the 17978 and AB5075 *A. baumannii* strains that resulted in apparent virulence differences using the *G. mellonella* as a host (Gebhardt et al., 2015) could explain the observation that none of the A1S_0112-A1S_0119 genes were identified as virulence genes when tested using this experimental infection model, which proved the critical role iron acquisition plays in *A. baumannii*'s virulence (Gaddy et al., 2012).

The putative functions for proteins encoded by this quorum-sensing regulated operon have been previously described (Clemmer et al., 2011) and include the acyl carrier protein (ACP) A1S_0114 and the predicted four-domain nonribosomal protein synthetase (NRPS) protein A1S_0115 (Allen and Gulick, 2014; Drake et al., 2016). The crystal structures of the *A. baumannii* AB307-0294 A1S_0115 orthologs, which shares 97.6% identity with the cognate 17978 gene product showed that glycine and AMP ligands were bound to the adenylation domain of this protein (PDB ID 4ZXI; Drake et al., 2016). Furthermore, glycine had the largest substrate specificity of the 20 proteinogenic amino acids tested, suggesting it could be the natural substrate incorporated into the biosynthetic product (Drake et al., 2016). A1S_0112 encodes an acyl-CoA synthase/AMP-acid ligase, which resembles fatty acid ACP ligases that activate and transfer fatty acids to ACPs *via* acyl AMP intermediates. A1S_0114, which encodes a free-standing ACP that is likely to contribute a tethered intermediate to the NRPS system involved in the biosynthesis of a secondary metabolite (Allen and Gulick, 2014), is the likely target for modification by A1S_0112, possibly yielding an acyl-ACP intermediate. A1S_0113, which encodes an acyl-CoA dehydrogenase (DH), is predicted to modify the intermediate product carried by the ACP or peptidyl carrier protein (PCP) domain of A1S_0115. The thioesterase domain (TE) of A1S_0115 is most likely responsible for the release of acyl/peptide chains from their covalent attachment to the ACP/PCP domains. A1S_0117 and A1S_0118 encode 424- and 621-amino acid proteins, respectively, that were annotated as hypothetical proteins of unknown function (Smith et al., 2007). A more detailed *in silico* analysis showed that A1S_0117 could be related to porins while A1S_0118 harbors two domains that resemble proteins in the epimerase/dehydratase and α/β hydrolase family, respectively. However, since proteins in these families have diverse functions, the function of A1S_0118 still could not be predicted. The A1S_0119 gene, the last component of this operon, encodes a predicted 254-amino acid phosphopantetheine transferase that is expected to perform this enzymatic conversion, which is the first step in making the ACP active and therefore the likely first step in NRPS biosynthesis by this operon. Finally, the A1S_0116 gene encodes a protein that belongs to the superfamily of resistance-nodulation-cell division (RND) transporters. The function of these pumps may include efflux of signaling molecules, as is the case for the *Pseudomonas* quinolone signal called PQS (Lamarche and Deziel, 2011), and thus A1S_0116 is potentially involved in the secretion of the secondary metabolite coded for by the A1S_0112-A1S_0119 polycistronic operon. All these predictions are in accordance with our experimental observation that the expression of A1S_0114 gene not only is associated with the virulence of 17978, but also required for the production of the Ac-505 secondary metabolite.

Our mass spectrometric analyses of a purified compound present in 17978 culture supernatants but absent from those obtained from the Δ0114 mutant showed that Ac-505 resembles a three-amino acid lipopeptide, but with non-standard linkages between the amino acids as well as to the hydrocarbon moiety. Typically NRPS-produced secondary metabolites have hydroxylated or non-hydroxylated acyl moieties attached by amide or ester (*N*- or *O*-linked) linkages, which was not observed with Ac-505. We propose that the acyl-chain moiety or the neighboring Gly-containing component of Ac-505 could have been modified by other NRPS enzymes. Since it is possible for adenylation domains to be used iteratively to add sequential amino acids, it is conceivable that two or more Gly residues were incorporated via the A1S_0115 adenylation domain into this part of Ac-505. The Ac-505 second and third amino acids are Cys and Gly residues linked via a standard peptide linkage although they are connected to the Gly-containing moiety through the sulfur group of the Cys side chain forming a thioether bridge. Since we saw no evidence for unsaturation of the acyl chain, it is possible that the A1S_0113 DH acts in *cis* to modify the growing product on the glycine-containing moiety bound to the PCP of A1S_0115 rather than acting on an acyl-moiety of an acyl-ACP. The precursor to the α-thio bond could be an epoxide or a cyclic compound, like a lactone. There are other NRPS-synthesized compounds that contain the so-called sactibiotic α-thio linkages such as the bacteriocins. These antimicrobial peptides contain a linkage between a cysteine thiol and the α-carbon of another amino acid residue. In the case of Ac-505, it is not clear how the α-thiol bond would be formed.

It is of note that the Ac-505 Cys-Gly moiety is potentially derived from glutathione, a tripeptide (L-γ-glutamyl-L-cysteinyl-glycine) that is found in high concentrations intracellularly in *A. baumannii* and is linked with increased resistance to antibiotics (Kwon et al., 2013). Glutathione is known to deactivate xenobiotics *via* conjugation in order to make them less toxic. Glutathione conjugation most commonly occurs via a nucleophilic attack by the glutathione cysteinyl thiol on an electrophilic carbon such as a lactone or epoxide (Wang and Ballatori, 1998). The γ-glutamyl residue can then be cleaved by a γ-glutamyl transpeptidase localized outside the plasma membrane, leaving the conjugated Cys-Gly, as occurs in the case of the glutathione-modified microcystin (Schmidt et al., 2014), a cyanobacteria toxin and intra- and extra-cellular signaling molecule (Makower et al., 2015).

The Ac-505 that we detected both inside and outside 17978 cells, may be the result of the glutathione-mediated de-activation of a secondary metabolite. This potential precursor to Ac-505 would contain the hydrocarbon-chain and modified glycine-moiety and should have an electrophilic carbon, but the exact structure is unknown. Modification of this precursor by glutathione could explain our observation that no active fraction of the spent media would restore the wild type phenotype to the Δ0114 mutant, even with highly purified Ac-505 (data not shown). A similar outcome was obtained during analysis of the *A. nosocomialis* M2 strain and the isogenic derivatives M2-2 and M2-11, which harbor transposon insertions within the A1S_0113 and A1S_0115 orthologs, respectively, and display reduced surface motility (Clemmer et al., 2011). It is also possible that a different precursor to Ac-505 with a conjugated tri-peptide glutathione (prior to cleavage of the γ-glutamyl moiety), could be the active compound. We saw no evidence in the LC-MS analysis of spent media for the presence of a glutathione-conjugated derivative and were not able to identify any Ac-505 lipophilic precursors.

In summary, our work provides novel evidence that the A1S_0114 gene positively affects *A. baumannii* biofilm biogenesis, adherence and virulence responses. The molecular and cellular mechanism by which these responses are achieved and whether Ac-505 is the active effector responsible for these responses are issues that will remain unknown until this small secondary metabolite and its biosynthetic pathway are fully understood. Such knowledge would provide novel insights into the pathobiology of *A. baumannii* and potentially facilitate the development of alternative tools needed for the treatment of infections caused by MDR isolates.

## Author contributions

SR: obtained the isogenic derivative and complemented strain and performed the qRT-PCR and attachment assays. AP: performed the scanning microscope assays and analyzed data of all work. TR: performed the LC-MS and high resolution MS and MS^*n*^ analysis. LÁ: performed the attachment assays. JV: performed the *C. elegans* and the mice virulence assays. AB: performed the mice virulence assays. EO: performed the scanning microscope experiments. BA: performed the *G. mellonella* virulence assay. MM: performed the attachment assays. SF: performed the complemented mutant strain analysis. MK: analyzed and supervised the work done in the Department of Chemistry and Biochemistry, Miami University, USA. LA: analyzed and supervised the work done in Department of Microbiology, Miami University, USA, interpreted all data and wrote the manuscript. GB: analyzed and supervised the work done in Spain. MP: coordinated and analyzed all the experiments of the work, interpreted data and wrote the manuscript.

## Funding

This work has been funded by Projects PI15/00860 to GB, CP13/00226 to AB, PI11/01034 to MP and P14/000059 to MP and AB, all integrated in the National Plan for Scientific Research, Development and Technological Innovation 2013-2016 and funded by the ISCIII—General Subdirection of Assessment and Promotion of the Research—European Regional Development Fund (FEDER) “A way of making Europe.” Miami University Research Funds from the departments of Microbiology and Chemistry and Biochemistry as well as funds from the College of Arts and Science Dean's office supported this work. We also want to thank the Spanish Network for Research in Infectious Diseases (REIPI RD12/0015/0014 to GB), co-financed by the European Development Regional Fund (EDRF) “A Way to Achieve Europe, Instituto de Salud Carlos III, Subdirección General de Redes y Centros de Investigación Cooperativa, Ministerio de Economía y Competitividad. JV was financially supported by the Sara Borrell Programme (ISCIII, Spain CD13/00373). SR was financially supported by the Agustí Pumarola Grant (Societat Catalana de Malalties Infeccioses i Microbiologia Clínica, SCMIMC) and Sociedad Española de Enfermedades Infecciosas y Microbiología Clínica (SEIMC). AP was financially supported by the Galician Plan for Research, Innovation and Growth (I2C Plan 2012-2016).

### Conflict of interest statement

The authors declare that the research was conducted in the absence of any commercial or financial relationships that could be construed as a potential conflict of interest.

## Supplementary material

Figure S1 **Confirmation of the polycistronic nature of the ***A. baumannii*** ATCC 17978 A1S_0112-A1S_0119 operon**. Total DNA (lanes 2–7), cDNA (lanes 8–13) and total RNA (lanes 14–19) samples were used as templates in PCR reactions using primers annealing to the A1S_0112-A1S 0113 (lanes 2, 8, and 14), A1S_0113-A1S_0115 (lanes 3, 9, and 15), A1S_0115-A1S_0116 (lanes 4, 10, and 16), A1S_0116-A1S_0117 (lanes 5, 11, and 17), A1S_0117-A1S_0118 (lanes 6, 12, and 18), and A1S_0118- A1S_0119 (lanes 7, 13, and 19) intergenic regions. Lanes 1 and 20 show the molecular weight standard Gene Ruler 1-Kb plus (Thermofisher Scientific). Molecular weight of each amplicon is indicated at the bottom of the figure.

Figure S2 **Growth of the 17978 parental and the Δ0114 isogenic deletion derivative strains**. The OD_600_values of each strain grown in LB or SB at 37°C for 24 h with shaking were determined hourly. Error bars represent the standard error (SE) of the mean.

Figure S3 **Ac-505 MS/MS spectra. (A)** MS data collected on the FT-ICR with ECD fragmentation in positive ion mode. **(B)** MS/MS collected on a MaXis QTOF in positive ion mode (LC-MS) and **(C)** negative ion mode (direct injection).

Figure S4 **MS/MS—based fragmentation predictions (A,B)** are two of the 40 total MS^*n*^ spectra from MS(3) to MS(6) recorded on the OSU Bruker amaZon in positive ion mode using direct injection of a HPLC purified Ac-505. MS(2) for 488.3 and MS(4) for 235.2 *m/z* fragment ions are shown in **(A,B)**, respectively. Neutral losses are shown in black. Panel **(C)** is a schematic diagram of MS/MS fragmentation of Ac-505 in positive ion mode and **(D)** is for negative ion mode.

Figure S5 **Predicted product ions of Ac-505 based on accurate mass and typical bond cleavage patterns under electrospray conditions in positive ion mode**.

Figure S6 **Predicted product ions of Ac-505 based on accurate mass and typical bond cleavage patterns under electrospray conditions in negative ion mode**.

Table S1 **Oligonucleotides used in the present study**.

Table S2 **HRMS data of Ac-505. Measured m/z-values are reported for MS fragment ions measured on ESI MaXis QTOF (LC-MS2) and 15T FT-ICR ECD (direct infusion) instruments in positive ion mode, respectively**. The molecular formulas of the product ions are based on accurate mass and isotopic pattern matching of the ions as well as typical bond cleavage patterns for electrospray ionization.

Table S3 **HRMS data of Ac-505 and MS2 fragments measured on a ESI MaXis QTOF (direct infusion) in negative ion mode**.
