# Supplementary figures and images for: Contribution of the A. baumannii A1S_0114 Gene to the Interaction with Eukaryotic Cells and Virulence
Source: Front Cell Infect Microbiol. 2017 Apr 3;7:108. doi: 10.3389/fcimb.2017.00108 (PMC5376624; doi:10.3389/fcimb.2017.00108)

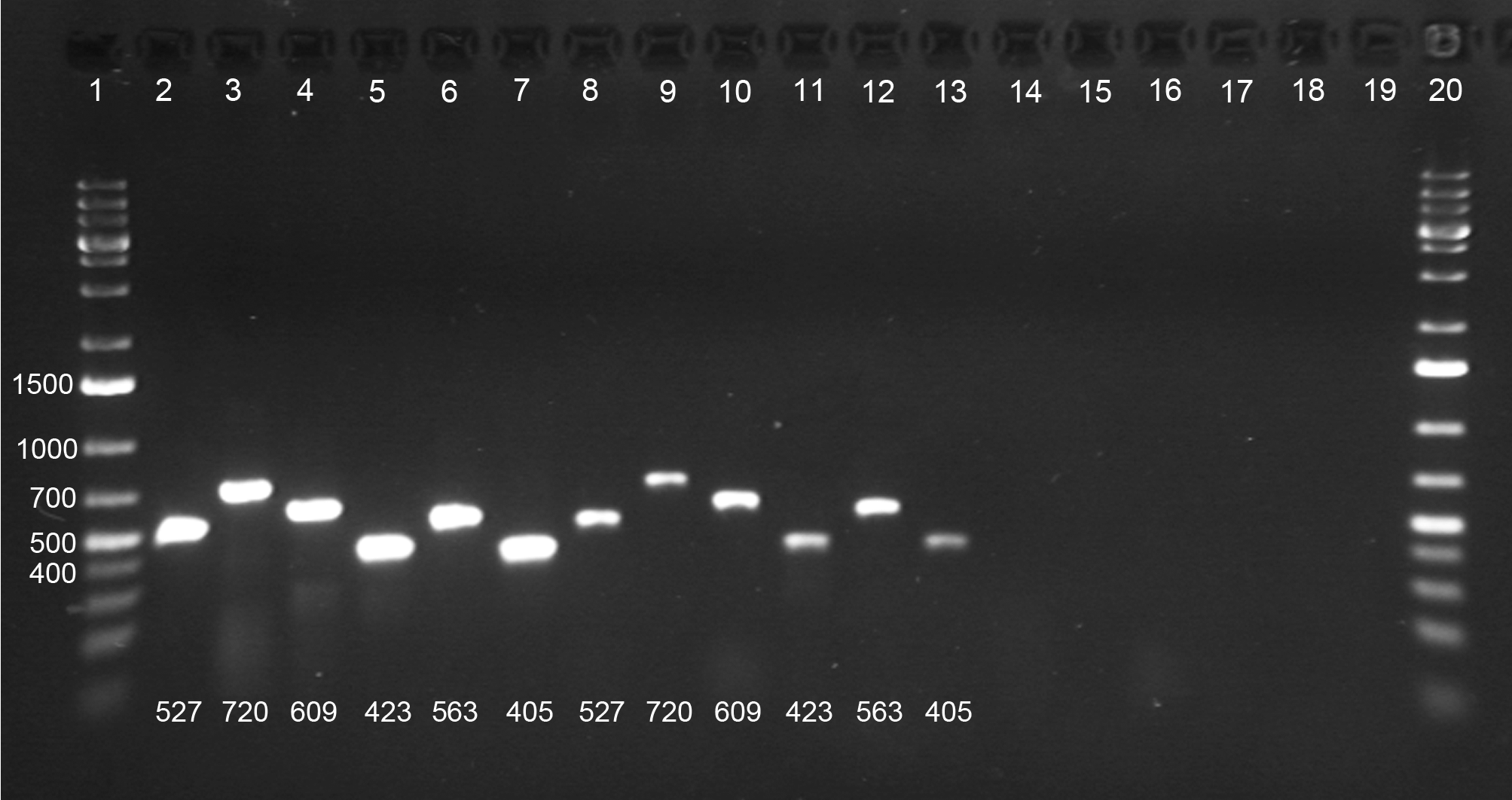

Supplement: Figure S1 — Confirmation of the polycistronic nature of the A. baumannii ATCC 17978 A1S_0112-A1S_0119 operon. Total DNA (lanes 2–7), cDNA (lanes 8–13) and total RNA (lanes 14–19) samples were used as templates in PCR reactions using primers annealing to the A1S_0112-A1S 0113 (lanes 2, 8, and 14), A1S_0113-A1S_0115 (lanes 3, 9, and 15), A1S_0115-A1S_0116 (lanes 4, 10, and 16), A1S_0116-A1S_0117 (lanes 5, 11, and 17), A1S_0117-A1S_0118 (lanes 6, 12, and 18), and A1S_0118- A1S_0119 (lanes 7, 13, and 19) intergenic regions. Lanes 1 and 20 show the molecular weight standard Gene Ruler 1-Kb plus (Thermofisher Scientific). Molecular weight of each amplicon is indicated at the bottom of the figure. [file Image1.TIF]

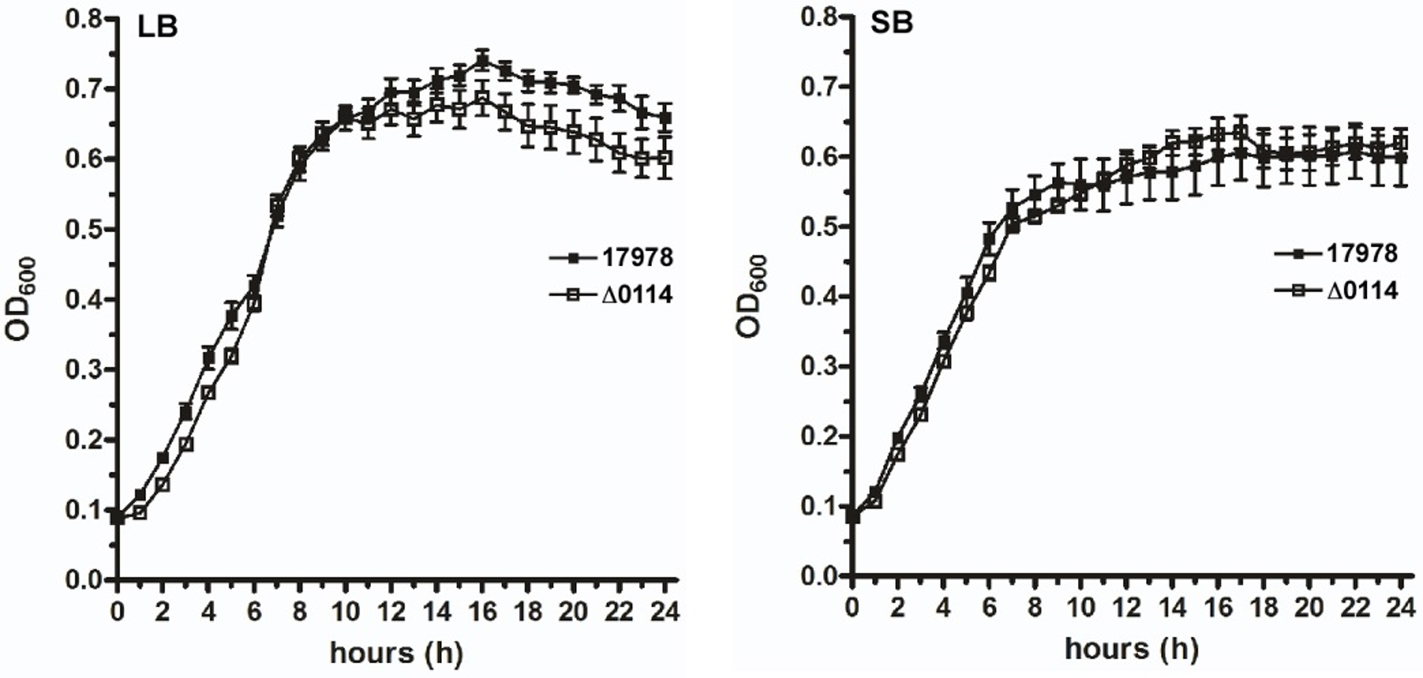

Supplement: Figure S2 — Growth of the 17978 parental and the Δ0114 isogenic deletion derivative strains. The OD600values of each strain grown in LB or SB at 37°C for 24 h with shaking were determined hourly. Error bars represent the standard error (SE) of the mean. [file Image2.TIF]

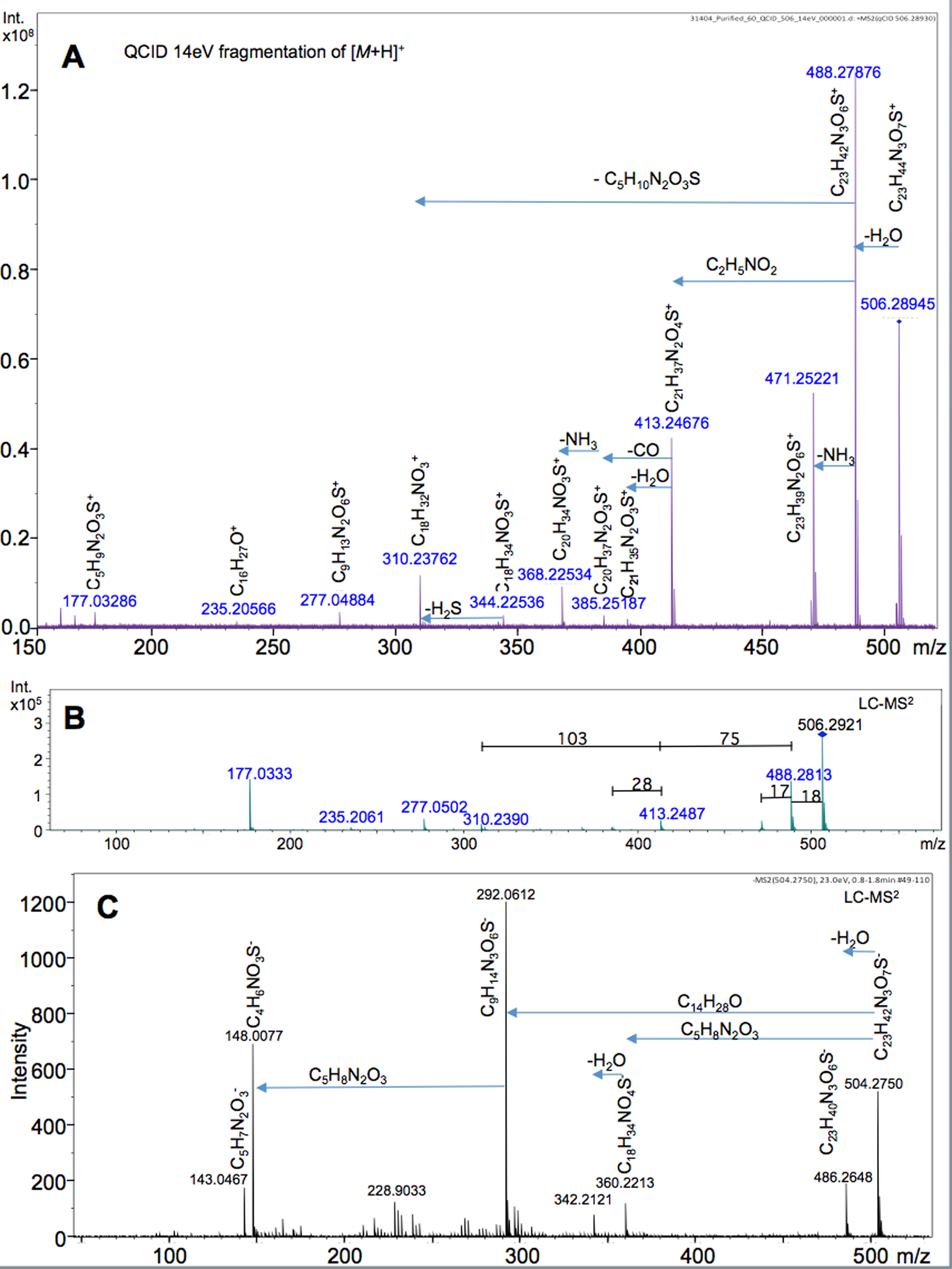

Supplement: Figure S3 — Ac-505 MS/MS spectra. (A) MS data collected on the FT-ICR with ECD fragmentation in positive ion mode. (B) MS/MS collected on a MaXis QTOF in positive ion mode (LC-MS) and (C) negative ion mode (direct injection). [file Image3.TIF]

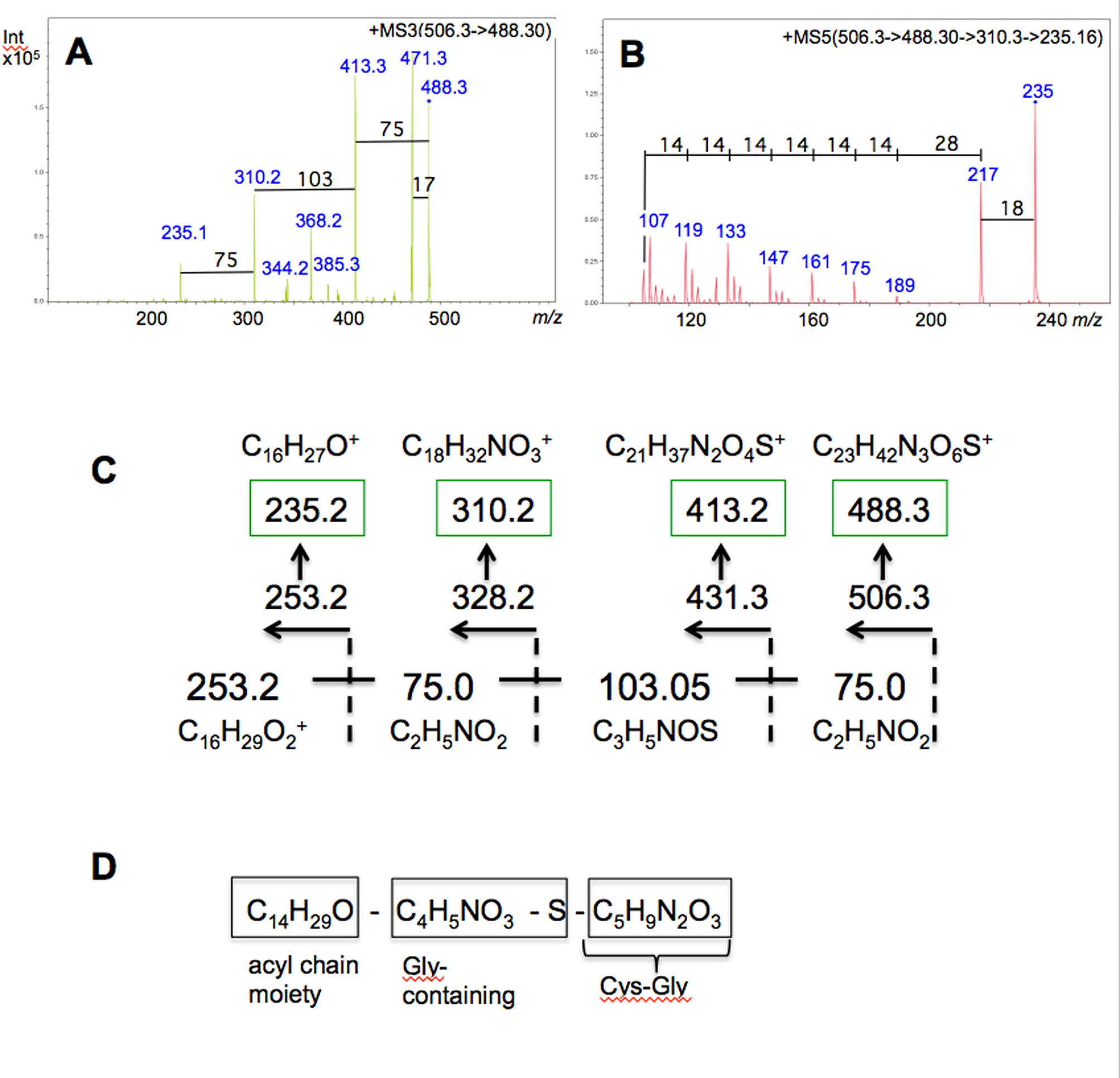

Supplement: Figure S4 — MS/MS—based fragmentation predictions (A,B) are two of the 40 total MSn spectra from MS(3) to MS(6) recorded on the OSU Bruker amaZon in positive ion mode using direct injection of a HPLC purified Ac-505. MS(2) for 488.3 and MS(4) for 235.2 m/z fragment ions are shown in (A,B), respectively. Neutral losses are shown in black. Panel (C) is a schematic diagram of MS/MS fragmentation of Ac-505 in positive ion mode and (D) is for negative ion mode. [file Image4.TIF]

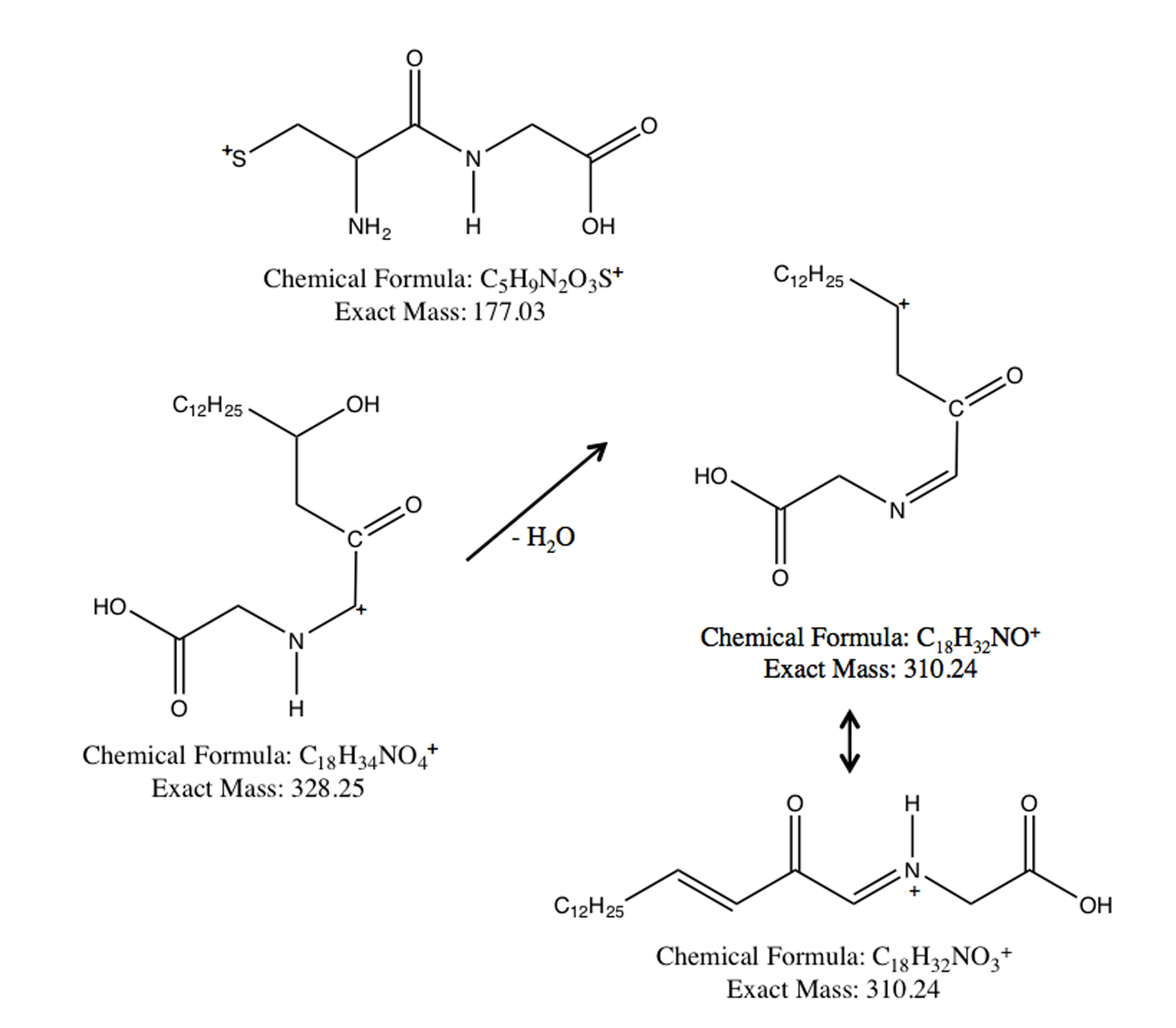

Supplement: Figure S5 — Predicted product ions of Ac-505 based on accurate mass and typical bond cleavage patterns under electrospray conditions in positive ion mode. [file Image5.TIF]

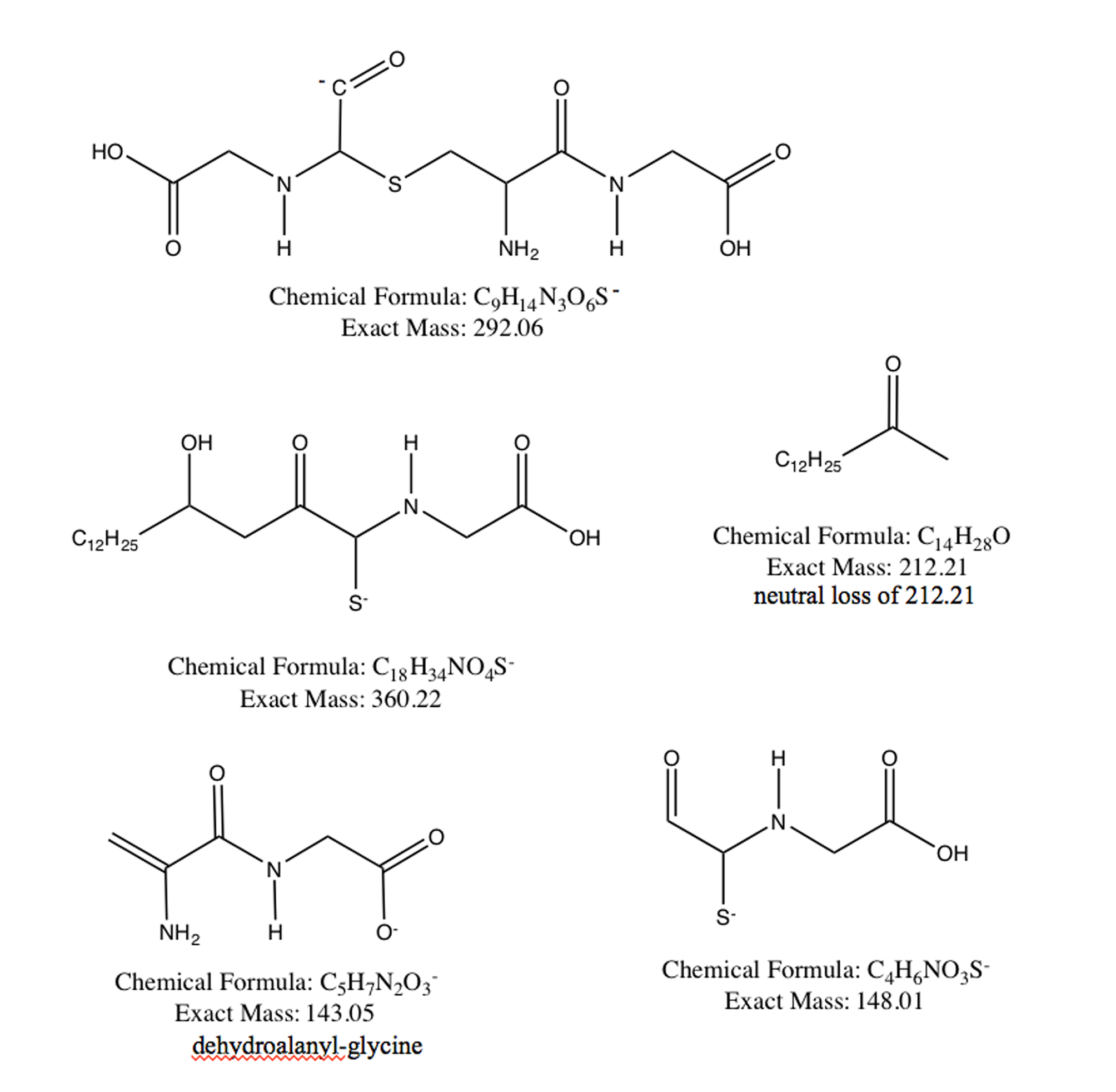

Supplement: Figure S6 — Predicted product ions of Ac-505 based on accurate mass and typical bond cleavage patterns under electrospray conditions in negative ion mode. [file Image6.TIF]
