# Supplementary material for: Contribution of the A. baumannii A1S_0114 Gene to the Interaction with Eukaryotic Cells and Virulence
Source: Front Cell Infect Microbiol. 2017 Apr 3;7:108. doi: 10.3389/fcimb.2017.00108 (PMC5376624; doi:10.3389/fcimb.2017.00108)
Supplement: Table S1 — Oligonucleotides used in the present study. [file Table1.DOCX]

**Table S1**. Oligonucleotides used in the present study

| Primer name | Sequence (5’-3’) | Purpose |
| --- | --- | --- |
| 0114UpF*Pst*I | CCCCTGCAGGGGTTGGTACGTGAGCAACTC | Construction of Δ0114 |
| 0114UpR*Eco*RI | GGGGAATTCCCCGCGCTCCAGTAAGCTTT | Construction of knockout strain Δ0114 |
| 0114DownF*Eco*RI | CCCGAATTCGGGGAGCCAACACTATTATGGGA | Construction of Δ0114 |
| 0114DownR*Bam*HI | GGGGGATCCCCCTCTCATAATCTTTCGCCAAG | Construction of Δ0114 |
| 0114extF | CAAGGAGTTTGAAACGAT | Confirmation of Δ0114 |
| 0114extR | CTCGCAGCAATAGACCAA | Confirmation of Δ0114 |
| 0114*Eco*RVF | CCCGATATCGGGACCGGTTAAAAAGGAGATTAC | Complementation of 17978 Δ114 |
| 0114*Bam*HIR | GGGGGATCCCCCCTGGTTCTAGTCGTGCAA | Complementation of 17978 Δ114 |
| pWH1266Fw | TAGGCTTGGTTATGCCGGTA | Confirmation the complementation of the 17978Δ0114 |
| pWH1266Rv | AAGGAGCTGACTGGGTTGAA | Confirmation the complementation of the 17978Δ0114 |
| KanaR*Pst*IFw | CCCCTGCAGGGGCCGGAATTGCCAGCTGGGGCG | Cloning the kanamycin resistance gene into pWH1266 plasmid |
| KanaR*Pst*IRv | GGGCTGCAGCCCTCAGAAGAACTCGTCAAGAAG | Cloning the kanamycin resistance gene into pWH1266 plasmid |
| 0112F | TTACTGCACCAAGGCCGAAT | Check expression of the operon |
| 0113R | AATTTCCATGCGACCTCCGA | Check expression of the operon |
| 0113F | GCTCGTATTGCTGTGTTGGG | Check expression of the operon |
| 0115R | CTTACGCGCAGTAGCGGATA | Check expression of the operon |
| 0115F | CCTCTCGATGCAGACCCATC | Check expression of the operon |
| 0116R | CGTACCTTCCGGATGTGGTT | Check expression of the operon |
| 0116F | CCGATATCCGTCCTTACGGC | Check expression of the operon |
| 0117R | GGGTTTACAGTGTGGTCCGT | Check expression of the operon |
| 0117F | GAGCTATGCTGCGTTATGCG | Check expression of the operon |
| 0118R | TTGTCTGGGCGTCCGATAAT | Check expression of the operon |
| 0118F | CTGGCGCAGGTCATAATCCA | Check expression of the operon |
| 0119R | AATAAGGTCCGCGGAGTGAC | Check expression of the operon |
| csuA/B F | GCAGCTGTTACTGGTCAG | qRT-PCR |
| csuA/B R | GTCTGTGCGTTCACCACC | qRT-PCR |
| A1S_2091 F | GTCCACCATCAAATGACAAAGTCC | qRT-PCR |
| A1S_2091 R | CTGTGTCCTGAATACCTCAGC | qRT-PCR |
| A1S_1510 F | GATGTTGCTGGTCGTACACC | qRT-PCR |
| A1S_1510 R | GACATTGGTAGCTGCACCAG | qRT-PCR |
| A1S_0690 F | AAACAACCGCAACTCGTGG | qRT-PCR |
| A1S_0690 R | CAGCGGCGTCTTTAATACC | qRT-PCR |
| ompA F | CGACGCTTTATCTCTTCG | qRT-PCR |
| ompA R | GGAGCAGCAGGCTTGAAG | qRT-PCR |
| recA F | TACAGAAAGCTGGTGCATGG | qRT-PCR |
| recA R | TGCACCATTTGTGCCTGTAG | qRT-PCR |
