# Supplementary material for: Contribution of the A. baumannii A1S_0114 Gene to the Interaction with Eukaryotic Cells and Virulence
Source: Front Cell Infect Microbiol. 2017 Apr 3;7:108. doi: 10.3389/fcimb.2017.00108 (PMC5376624; doi:10.3389/fcimb.2017.00108)
Supplement: Table S2 — HRMS data of Ac-505. Measured m/z-values are reported for MS fragment ions measured on ESI MaXis QTOF (LC-MS2) and 15T FT-ICR ECD (direct infusion) instruments in positive ion mode, respectively. The molecular formulas of the product ions are based on accurate mass and isotopic pattern matching of the ions as well as typical bond cleavage patterns for electrospray ionization. [file Table2.DOCX]

**Table S2**. HRMS data of Ac-505. Measured *m/z* values are reported for MS fragment ions measured on ESI MaXis QTOF (LC-MS^2^) and 15T FT-ICR ECD (direct infusion) instruments in positive ion mode, respectively. The molecular formulas of the product ions are based on accurate mass and isotopic pattern matching of the ions as well as typical bond cleavage patterns for electrospray ionization.
