# Supplementary material for: Contribution of the A. baumannii A1S_0114 Gene to the Interaction with Eukaryotic Cells and Virulence
Source: Front Cell Infect Microbiol. 2017 Apr 3;7:108. doi: 10.3389/fcimb.2017.00108 (PMC5376624; doi:10.3389/fcimb.2017.00108)

**Table S3**. HRMS data of Ac-505 and MS^2^ fragments measured on a ESI MaXis QTOF (direct infusion) in negative ion mode.


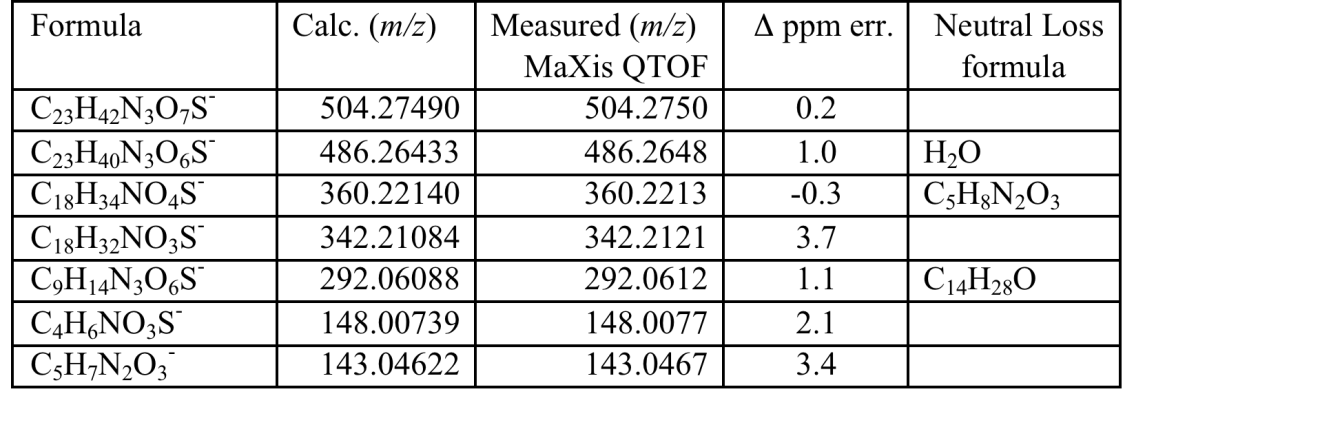

Supplement: Table S3 — HRMS data of Ac-505 and MS2 fragments measured on a ESI MaXis QTOF (direct infusion) in negative ion mode. [file Table3.DOCX]
